# Rhabdomyolysis in a 10-month-old child: A case report and literature review

**DOI:** 10.1097/MD.0000000000050012

**Published:** 2026-08-21

**Authors:** Hui Shao, Na Wang

**Affiliations:** a Department of Pediatrics, Wuhan No.1 Hospital, Wuhan, China.

**Keywords:** acute kidney injury, creatine kinase, diagnosis, infant, rhabdomyolysis

## Abstract

**Rationale:**

Early diagnosis and prompt treatment of rhabdomyolysis (RM) in children are crucial to prevent potential complications, such as acute renal failure (ARF). Given that the clinical manifestations of RM in pediatric patients are often atypical, especially in infants, this case report aims to improve the recognition of RM by analyzing a specific clinical case and reviewing relevant literature.

**Patient concerns:**

A 10-month-old girl was admitted to the hospital with a 3-day history of fever and a 1-day history of cough and rash. The child had no obvious cause of fever and showed no typical symptoms of RM, such as myalgia or myoglobinuria.

**Diagnoses:**

Physical examination revealed red papules on the skin of the whole body. Laboratory tests showed a significant increase in serum creatine kinase (CK; 119,985 IU/L) and myoglobin (816.7 µg/L). The diagnosis was confirmed as RM based on these laboratory results, despite the absence of the classic clinical triad.

**Interventions:**

The patient received symptomatic and supportive treatments, including atomization with budesonide and ipratropium bromide to relieve cough, oral administration of cefaclor to resist infection, and sodium bicarbonate to alkalize urine and prevent renal damage. In addition, methylprednisolone sodium succinate was used to regulate immunity, along with hepatoprotective agents (reduced glutathione) and myocardial nutrition agents (creatine phosphate sodium).

**Outcomes:**

After 13 days of hospitalization, the patient’s symptoms (rash, cough, and thrush) disappeared. Laboratory parameters, including CK, CK isoenzyme, liver enzymes, and myoglobin, decreased significantly and returned to normal levels during follow-up. No ARF or recurrence was observed during the 6-month follow-up period.

**Lessons:**

The recognition of RM in children should be improved. Clinicians should be vigilant when encountering atypical symptoms in conjunction with substantially elevated serum CK. Early and accurate diagnosis, along with active fluid resuscitation, can effectively reduce the incidence of ARF and improve prognosis.

## 1. Introduction

Rhabdomyolysis (RM) refers to a group of clinical syndromes caused by various reasons, such as the destruction and disintegration of rhabdomyosin, which makes intracellular components such as creatine kinase (CK) and myoglobin enter the blood circulation, causing internal environment disorder and tissue and organ damage.^[1,2]^ The most common complication of RM is acute renal failure (ARF) due to acute tubular necrosis resulting from mechanical obstruction by myoglobin, particularly if the serum CK > 16,000 IU/L. Patients with ARF have a significantly higher mortality rate. Thus, timely recognition of RM is vital for treatment.^[3]^

RM is a typical clinical triad characterized by myalgia, muscle weakness, and pigmenturia. The clinical manifestations of RM in children are related to age. It is more atypical when the age is younger, making it a challenging diagnosis in pediatric management.^[4,5]^ This paper summarizes a clinical case of a child with RM in our department and analyzes its clinical characteristics with a literature review.

## 2. Case presentation

A 10-month-old girl was admitted to a hospital, presented with fever for 3 days and cough and rash for 1 day. The child had no obvious cause of fever. The highest temperature was 38.3°C, with irregular fever, no chills, no nasal congestion, runny nose, or sneezing, no skin petechiae, no vomiting, diarrhea, mucus, or purulent stool, and no convulsions, lethargy, or coma. After the illness started, she was given pediatric paracetamol orally by her family for 1 day, but the fever did not improve. She was brought to the hospital the next day and treated with antibiotic cephalosporin and Chinese patent medicine orally for 2 days. Her body temperature returned to normal, and no fever reoccurred.

Three days later, the child had a cough, which was characterized by a single cough, sticky phlegm, and a rash. The next day, she was brought to the outpatient department in our hospital. On examination, there were red papules the size of rice grains on the skin of the whole body, with no cyanosis, dyspnea, or other symptoms. She was treated with Chinese medicine “Lanqin oral liquid” orally for 1 day. Two days later, she revisited the outpatient department for further diagnosis and treatment. The routine blood test results showed white blood cell: 14.65 × 10^9^/L, red blood cell (RBC): 5.36 × 10^12^/L, hemoglobin: 142 g/L, mean RBC volume: 77.4 fL, mean hemoglobin level: 26.5 pg, total platelet count: 325 × 10^9^/L, neutrophil: 36.3%, hypersensitivity C-reactive protein (CRP): 3.6 mg/L, and CRP: <5.0 mg/L.

The child was then admitted to our department for acute bronchitis and thrush. She had no obvious past medical or surgical history and no history of trauma or blood transfusion. On admission, physical examination revealed a temperature of 36.1°C, heart rate of 117 beats per minute, respiratory rate of 28 breaths per minute, and peripheral capillary oxygen saturation of 99% on room air. The patient weighed 8 kg, was alert but slightly lethargic, had unlabored breathing, and exhibited no signs of respiratory distress (e.g., no tracheal tug, intercostal, or subcostal retractions). On examination, many red papules the size of rice grains could be seen on the skin of the whole body, and the skin between the papules was normal. No scratches, facial or perioral cyanosis, or palpable superficial lymph nodes were noted on physical examination. Breath sounds were coarse bilaterally, with rhonchi audible at the lung bases. The heart rate was 117 beats per minute, with a regular rhythm. The heart sounds were normal, and no murmur was heard in the auscultation areas of all valves. The abdomen was flat and soft, the liver and spleen were not palpable, the percussion was drum sounds, and the bowel sounds were 4 times per minute. Peripheral perfusion was adequate, and muscle strength and tone in all 4 limbs were normal. The pathological signs were negative. The initial diagnosis was acute bronchitis, thrush, and viral rash.

After admission, budesonide and ipratropium bromide were atomized to relieve cough and eliminate phlegm, and a sodium bicarbonate diluted solution was used to scrub the mouth. The electrocardiogram was unremarkable, and procalcitonin, CRP, and stool studies were within normal limits. The respiratory bacterial and virus antibodies (*Legionella pneumophila* serotype 1 IgM, *Mycoplasma pneumoniae* IgM, Q-fever rickettsia IgM, *Chlamydia pneumoniae* IgM, adenovirus IgM, respiratory syncytial virus IgM, influenza A virus IgM, influenza B virus IgM, and parainfluenza virus IgM) were all negative. The coagulation profile, renal function, and urinalysis (including urine protein) were within normal limits. The results of other laboratory tests are shown in Table 1.

**Table 1:** Results of the laboratory tests after admission.

Index	D1^▲^	D2	D3	D4	D6	D9	D12
White blood cell (×10^9^/L)	15.50	–	–	9.99	–	–	–
Red blood cell (×10^12^/L)	5.28	–	–	4.67	–	–	–
Hemoglobin (g/L)	143	–	–	126	–	–	–
Neutrophil (×10^9^/L)	2.53	–	–	2.78	–	–	–
Lymphocytes (×10^9^/L)	10.14	–	–	3.08	–	–	–
Creatine kinase (IU/L)	119,985	>1600*	19,246	9861	3035	1999	822
Creatine kinase isoenzyme (IU/L)	346	>300*	114	84	44	47	34
Lactate dehydrogenase (IU/L)	3026	>2150*	854	699	447	355	326
α-hydroxybutyrate dehydrogenase (IU/L)	1563	–	542	469	329	261	236
Alanine aminotransferase (IU/L)	1277	1042	727	597	393	196	105
Aspartate amino transferase (IU/L)	2672	1464	633	371	133	90	64
Potassium (mmol/L)	6.5	4.6	–	4.6	5.4	5.1	5.2
Natrium (mmol/L)	138.1	137.6	–	141.6	140.1	137.1	136.3
Urea nitrogen (mmol/L)	4.2	2.7	–	2.0	3.6	3.6	4.7
Creatinine (µmol/L)	22	21	–	22	18	21	21
Uric acid (µmol/L)	224	165	–	175	133	206	225
Myoglobin concentration (µg/L)	–	816.7	–	–	–	464.7	154.9
Urinary albumin	–	–	–	–	–	–	–
Urine occult blood	–	–	–	–	–	–	–

The second myocardial enzyme spectrum examination is an urgent investigation item in our hospital, and the rest are general investigation items. The detection range of the urgent investigation instruments and equipment is relatively narrow, so the reported value is higher than the upper limit. D1^▲^ indicates the first day of admission. The symbol “−” denotes not tested.

* Values indicate the maximum measurable range of the assay instrument.

Reduced glutathione was administered intravenously upon the availability of laboratory results for its hepatoprotective effects and to reduce elevated liver enzymes; creatine phosphate sodium was used to nourish the myocardial cells and protect important organs; methylprednisolone sodium succinate (2 mg/kg, twice a day, for 3–5 days) was used to regulate immunity and block the progress of the disease; sodium bicarbonate was used to alkalize urine and rehydrate to prevent renal damage caused by elevated CK, and cefaclor dry suspension was given orally to resist infection.

Concurrently, the differential diagnosis was refined to include RM, acute bronchitis, oral candidiasis (thrush), and viral exanthem. The serum myoglobin concentration was 816.7 µg/L, which was significantly increased. Relevant laboratory abnormalities, including elevated CK, were repeated, and the diagnosis was confirmed based on the test results.

On the third day of hospitalization, after symptomatic supportive treatment, the rash and cough symptoms disappeared, and the CK, CK isoenzyme, lactate dehydrogenase (LDH), α-hydroxybutyrate dehydrogenase (α-HBDH), alanine aminotransferase (ALT), and aspartate aminotransferase (AST) were all decreased significantly.

On the fourth day of hospitalization, the renal function, electrolytes, and blood routine were normal; the urine protein and urine occult blood were negative, and the CK and CK isoenzyme were significantly lower than before. On the fifth day of hospitalization, the thrush disappeared.

On the sixth and ninth days of hospitalization, the CK and CK isoenzyme were significantly lower than before, and the renal function and electrolytes were normal.

On the ninth day of hospitalization, the serum myoglobin concentration and CK were significantly lower than before.

On the 12th day of hospitalization, the CK was 822 IU/L, CK isoenzyme was 34 IU/L, LDH was 326 IU/L, α-HBDH was 236 IU/L, ALT was 105 IU/L, AST was 64 IU/L, myoglobin concentration was 154.9 µg/L (normal), and the renal function and electrolytes were rechecked.

On hospital day 13, the patient was discharged at the family’s request due to financial constraints and personal circumstances.

Two weeks after discharge, liver function tests, cardiac enzymes, renal function tests, and urinalysis were all within normal limits. No recurrence was observed during the 6-month follow-up period.

## 3. Discussion and conclusions

With the increasing clinical reports of RM in recent years, clinicians have widely recognized the diversity and complexity of its etiology, clinical manifestations, laboratory tests, and complications. Clinical cases of RM in children are relatively rare, and its incidence in children is only 0.26%.^[6]^ Serum CK is the most specific index to diagnose RM. Usually, the increase of serum CK is 5 to 10 times higher than the normal upper limit, accompanied by obvious muscle pain and soy sauce urine, which can be diagnosed as RM. Excessive exercise, squeezing, trauma, adverse drug reactions, infection, immune diseases, and congenital metabolic diseases may all lead to RM.^[7,8]^

According to the current research, RM can be divided into traumatic and nontraumatic cases, and the present case is nontraumatic RM. The child was admitted with a 3-day history of fever and a 1-day history of cough and rash. RM was likely triggered by infection – an etiology less commonly reported than trauma (e.g., strenuous exercise) or adverse drug reactions. The main cause of RM in children is nontraumatic factors, among which infection is the most important cause.^[9]^ The possible mechanisms of infection leading to RM in children include direct injury of muscle cells caused by viruses and bacteria, inflammatory reactions caused by infection and fever, and adverse reactions caused by antibacterial and antiviral drugs. In addition, the muscle cells can be damaged if the infection involves the central nervous system with convulsions.^[9,10]^

On the first day of admission, the laboratory tests showed that the serum CK was 119,985 IU/L, CK isoenzyme was 346 IU/L, LDH was 3026 IU/L, α-HBDH was 1563 IU/L, ALT was 1277 IU/L, and AST was 2672 IU/L, all of which were significantly increased, especially CK. The increase of CK is an important and sensitive index in the diagnosis of RM, which generally starts to increase 2 to 12 hours after muscle injury, reaches the peak in 1 to 3 days, and begins to decrease after 3 to 5 days.^[11]^ The serum CK of this child accords with the above rules. At the same time, myalgia and myoglobinuria are also important bases for the diagnosis of RM, but this child has no pain manifestations such as muscle squeezing and crying, and there is no myoglobinuria. Serum CK and serum myoglobin concentrations of this child are obviously increased, which shows that myoglobinuria and muscle pain are not necessary conditions for the diagnosis of RM.^[12,13]^

The most serious complication of RM is ARF, and its mechanisms include renal vascular contraction, which reduces the glomerular filtration rate, cast formation, and a large amount of myoglobin excretion leading to renal tubular blockage and ischemia, which further promotes the toxic effect of myoglobin on renal tubules. After muscle injury, the inflammatory exudation of the local tissue increases, and a large amount of inflammatory fluid accumulates in the muscle tissue, which reduces the effective circulating blood volume and leads to renal ischemia. Inflammatory mediators produced by RM also lead to ARF and multiple organ dysfunction.^[14–16]^ According to clinical research, 10% to 50% of patients with RM have ARF, accounting for about 5% to 25% of patients with ARF.^[12]^ At present, there are many reports of ARF caused by RM in adults, but few in children. This child has no ARF, which may benefit from the timely blood test and fluid replacement after admission, and RM should be considered according to the results of the laboratory tests in a timely manner.

At present, the diagnosis of nontraumatic RM is mainly based on the following indicators: first, there are certain incentives and many infection factors in children; second, clinical manifestations include myasthenia, myalgia, and brown urine; third, serum CK > 1000 IU/L; fourth, the concentration of serum myoglobin is increased; fifth, urinary myoglobin is significantly increased; sixth, urinary occult blood is positive, and no RBCs are found under microscopic examination. Through the understanding of RM in children, it is found that the symptoms in this case are hidden and the clinical manifestations are atypical. It is necessary to have serum CK > 1000 IU/L to diagnose RM.^[12]^ Clinically, RM in children is rare, so it is necessary for first-line pediatricians to fully grasp the diagnostic criteria to diagnose RM as soon as possible, treat early, and reduce adverse prognosis.

## Author contributions

**Conceptualization:** Hui Shao, Na Wang.
